# Deletion of Ubiquitin Fold Modifier Protein Ufm1 Processing Peptidase Ufsp in *L. donovani* Abolishes Ufm1 Processing and Alters Pathogenesis

**DOI:** 10.1371/journal.pntd.0002707

**Published:** 2014-02-20

**Authors:** Sreenivas Gannavaram, Sonya Davey, Ines Lakhal-Naouar, Robert Duncan, Hira L. Nakhasi

**Affiliations:** Laboratory of Emerging Pathogens, Division of Emerging and Transfusion Transmitted Diseases, Center for Biologics Evaluation and Research, FDA, Bethesda, Maryland, United States of America; National Institute of Allergy and Infectious Diseases, United States of America

## Abstract

Previously, we showed *Leishmania donovani* Ufm1 has a Gly residue conserved at the C-terminal region with a unique 17 amino acid residue extension that must be processed prior to conjugation to target proteins. In this report, we describe for the first time the isolation and characterization of the *Leishmania* Ufm1-specific protease Ufsp. Biochemical analysis of *L. donovani* Ufsp showed that this protein possesses the Ufm1 processing activity using sensitive FRET based activity probes. The Ufm1 cleavage activity was absent in a mutant Ufsp in which the active site cysteine is altered to a serine. To examine the effects of abolition of Ufm1 processing activity, we generated a *L. donovani* null mutant of Ufsp (LdUfsp^−/−^). Ufm1 processing activity was abolished in LdUfsp^−/−^ mutant, and the processing defect was reversed by re-expression of wild type but not the cys>ser mutant in the LdUfsp^−/−^ parasites. Further LdUfsp^−/−^ mutants showed reduced survival as amastigotes in infected human macrophages but not as promastigotes. This growth defect in the amastigotes was reversed by re-expression of wild type but not the cys>ser mutant in the Ufsp^−/−^ indicating the essential nature of this protease for *Leishmania* pathogenesis. Further, mouse infection experiments showed deletion of Ufsp results in reduced virulence of the parasites. Additionally, Ufsp activity was inhibited by an anti-leishmanial drug Amphotericin B. These studies provide an opportunity to test LdUfsp^−/−^ parasites as drug and vaccine targets.

## Author Summary

Ubiquitin and ubiquitin like proteins (Ubls) and the enzymes that mediate the conjugation/deconjugation reactions have not been well studied in protozoan parasites despite their widely recognized importance in a broad range of cellular functions in eukaryotes. We have previously reported that Ufm1 has distinct protein targets and cellular localization in the human parasite *Leishmania donovani* and deletion of Ufm1 in *L. donovani* adversely impacts the pathogenesis suggesting that Ufm1 associated enzymes could be exploited as drug targets. Using sensitive FRET based activity probes we identified the Ufm1 processing peptidase Ufsp in *L. donovani*. In addition, we show that deletion of Ufsp specifically reduces the survival of amastigotes, the parasite stage that is present in the humans thus altering the pathogenesis. Studies showing inhibition of Ufsp activity by anti-leishmanial drug further suggests that *Leishmania* Ufsp can serve as a novel target for pharmacological intervention for this parasite that causes deadly disease.

## Introduction

Leishmaniasis is a spectrum of diseases caused by protozoan parasites belonging to several different *Leishmania* species. These blood borne pathogens are currently prevalent in 88 countries around the World with an estimated 2 million new cases each year [1]. At present there are no effective vaccines against any of the clinical forms of leishmaniasis. Further drugs against this parasite are becoming limited in their usefulness due to inappropriate use and because of the development of drug resistance against pentavalent antimonials [2]. Recent advances in genome sequencing ushered in post-genomic analysis of *Leishmania* parasites in terms of parasite biology in the sand fly vector and mammalian host, including host responses [3]. Yet, the parasitic factors involved in pathogenesis associated with any form of leishmaniasis remain to be fully understood, as the parasite virulence is determined by numerous factors.

Protein modifications by ubiquitin and ubiquitin-like proteins (Ubls) are widely described in eukaryotes [4]. The modification of target proteins by Ubls involves covalent attachment of Ubls to a substrate protein [5]. The best-known consequence of ubl conjugation is the targeting of proteins for degradation by the proteasome [6]. In addition to proteasomal targeting, conjugation by Ubls have been shown to affect a broad range of functions including subcellular localization, endocytosis, membrane trafficking, protein kinase activation, DNA repair, chromatin dynamics and protein-protein interactions [7]. Ubiquitin-fold modifier 1 (Ufm1) that possesses a similar tertiary structure compared to ubiquitin, has recently been identified as a novel protein-conjugation system [8]. Attachment of Ufm1 to its substrate proteins has been shown to follow enzymatic reactions commonly found in many ubl conjugation reactions. Ufm1 is synthesized as a precursor form and processed C terminally by two specific proteases, UfSP1 and UfSP2 in humans [9]. The processed Ufm1 is activated by the E1-like enzyme, Uba5, and then transferred to an E2 enzyme, Ufc1. Finally the Ufm1 is covalently conjugated to the substrate proteins via an E3-like enzyme Ufl1 [10]. Studies in mouse revealed that an ER protein named C20orf116, with unknown function is the substrate protein for mammalian Ufm1 [11], [12]. Although Ufm1 has been studied in humans, its functions are still not completely understood. Deletion of Uba5, the Ufm1 activating enzyme resulted in embryonic lethality in mice [12] suggesting that genetic manipulation of some of the Ufm1 associated proteins may not be feasible in mammalian cells.

We have recently shown that the human protozoan parasite *Leishmania donovani* contains the full complement of Ufm1 conjugation reactions that is revealed by the presence of substrate proteins conjugated to Ufm1 [13]. Among the substrate proteins conjugated to *Leishmania* Ufm1 is a mitochondrial trifunctional protein (MTP) involved in the β-oxidation of fatty acids [13], [14]. Deletion of Ufm1 from *L. donovani* resulted in loss of β-oxidation of fatty acids due to the absence of conjugation\ of MTP with Ufm1 [14]. These results suggested the importance of Ufm1 mediated conjugation in the physiology of *L. donovani*. In *L. donovani* Ufm1 must be processed to remove the 17-aminoacid C-terminal extension to expose the glycine residue important for the conjugation to occur indicating the presence of such processing activity in the parasite [13]. In this report, we have demonstrated the presence of Ufm1 processing activity of LdUfsp in *L. donovani* and this activity can be inhibited by antileishmanial compound Amphotericin B. Deletion of Ufsp affects this Ufm1 processing activity and results in reduced survival of amastigotes as was previously seen with Ufm1 deletion [14] further suggesting the importance of the role of Ufsp mediated Ufm1 processing in the parasite survival. Reduction in virulence of *Leishmania* Ufsp null mutant implies that this novel strain can have important applications as a live attenuated vaccine candidate as has been demonstrated by us and others using gene deleted *Leishmania* parasites and additionally aid identify drug candidates that target Ufsp and mimic the loss of Ufsp function in *Leishmania* [15]. To our knowledge *Leishmania* Ufsp1 null mutant is the first of its kind and Ufsp null mutant can be useful not only in the understanding of *Leishmania* pathogenesis but also in other organisms including humans.

## Methods

### Plasmids and parasite cultures

*Leishmania donovani* promastigotes (strain 1S, WHO designation: MHOM/SD/62/1S) were grown in M199 medium containing 10% heat inactivated fetal bovine serum. Promastigotes were transfected by electroporation and selected for growth in medium containing Nourseothricin (100 µg/ml). Recombinant Ufsp−/− parasites reexpressing wild type or mutant Ufsp were similarly selected in medium containing Geneticin (G418; 100 µg/ml). These drug-resistant cells were used in all subsequent experiments. Axenic amastigotes of wild type or the mutant lines were generated following a published protocol [16], [17].

### Preparation of fluorescent substrates

For the analysis of enzymatic activity encoded by the putative LdUfsp, FRET-based fluorescent probes were prepared following the methodology described by Tatham and Hay [18] with brief modifications. Briefly, coding sequences containing either wild type LdUfm1 or the mutant LdUfm1 in which C'terminal Gly is altered to Ala were amplified with flanking BamHI and HindIII sites. The resulting fragments were ligated into the BamHI-HindIII digested pHis-TEV-30a-YFP-SUMO-1-ECFP plasmid, thus replacing the SUMO-1. The ligation was confirmed by nucleotide sequencing of the bacterial expression plasmids. The plasmids were used to transform *E. coli* (BL21-PLysS, Invitrogen). Recombinant fusion proteins containing the wild type or the mutant LdUfm1 were prepared as described [18].

### Preparation of recombinant LdUfsp and anti-Ufsp antibodies

For preparing the recombinant protein encoded by the putative LdUfsp, the open reading frame corresponding to 427–1518 bp was PCR amplified and ligated into pEXP5-CT-Topo plasmid vector. The authenticity of the plasmid was confirmed by nucleotide sequencing. BL21-pLysS bacterial cells were transformed with this expression plasmid and the recombinant Ufsp protein was purified under native conditions. This recombinant protein was used for preparing polyclonal antibodies in rabbit (Spring Valley Labs, Sykesville, MD).

### Fractionation of *Leishmania* lysates for Ufm1processing activity

For fractionation of Ufm1 processing activity, extracts were prepared from the *Leishmania* cultures either wild type, Ufsp^−/−^ or Ufsp−/− parasites re-expressing Ufsp (2–3×10^9^ cells) in a buffer containing 25 mM Tris HCl pH 8.0, 150 mM NaCl, 1%NP-40. The extracts were passed through a strong anion exchange column (Q-column, Pierce Biotechnology) and fractions were collected in a buffer containing increasing salt concentration (0.2–2 M NaCl) following the protocol suggested by the manufacturer. The fractions were tested for the Ufm1 processing activity in a FRET based cleavage assay and the positive fractions were pooled. These pooled fractions were dialyzed against a buffer containing 25 mM Sodium acetate pH 5.5. These were passed through strong cation exchange column (S-column, Pierce Biotechnology) and fractions were collected in a buffer containing increasing salt concentration (0.2–2 M NaCl). The fractions were dialyzed against PBS and used in FRET based activity assays. Cell fractionation for localization studies was done using digitonin reagent as described previously [19].

### Enzymatic activity of recombinant LdUfsp

The activity of the recombinant LdUfsp was measured in an assay buffer containing 50 mM Tris-HCl, pH 7.5, 150 mM NaCl, 5 mM β-mercaptoethanol, 0.1 mg/ml bovine serum albumin (BSA). The recombinant enzyme (0.5 µg) and the FRET substrate (250 nM per well) were dispensed into the wells of a black 96 well clear bottom plate and followed by single readings at 480 and 530 nm at the start of the reaction and at the end of 60 minute incubation at 37°C in a Spectramax M5 fluorescent plate reader. The reduction in the fluorescence at 530 nm (Δ530) corresponding to YFP was measured after subtracting the reduction in 530 nm due to incubation at 37°C for 60 min in wells containing the probes alone. A reduction in the YFP fluorescence would indicate that the cleavage of Ufm1 has occurred and the emissions from the C′ terminal ECFP can no longer excite YFP fused at the N′ terminal end.

### Co-immunoprecipitation analysis

For co-immunoprecipitation analysis, 1×10^8^ *Leishmania* amastigotes were lysed in 1 ml of NET buffer (150 mM NaCl, 1 mM EDTA, 10 mM Tris–HCl, pH 7.5, 1% Nonidet P-40 with protease inhibitor cocktail) and the lysate was centrifuged at 12000 rpm for 20 min at 4°C to collect the supernatant. Five µl of anti-Ufsp antibodies were added to 500 µl to the lysate and the mixture was incubated under constant rotation overnight at 4°C. The complexes were immunoprecipitated with 25 µl of Protein-A Sepharose beads by incubation under constant rotation at 4°C for 1 hr. The precipitated complexes were washed five times with ice-cold NET buffer and eluted by boiling for 5 min in SDS sample buffer in the presence of β-mercaptoethanol. The supernatant was subjected to SDS–PAGE and analyzed by immunoblots with anti-Ufm1 antibodies.

### Macrophage infection

Human elutriated monocytes were resuspended at 1.8×10^5^ cells/ml in RPMI medium containing 10% FBS and macrophage colony-stimulating factor (20 ng/ml, ProSpec, Israel), plated at 0.5 ml/well on eight-chamber Lab-Tek tissue-culture slides (Miles Laboratories) and incubated for 9 days for differentiation into macrophages. The macrophage infection experiments were performed essentially as described earlier [19].

### Mouse infection studies

5- to 6-wk-old female BALB/c mice were infected via tail vein with 3×10^6^ stationary-phase wild type, LdUfsp^−/−^ or LdUfsp^−/−^+Ufsp^WT^ parasites. Infected mice were sacrificed after different periods of infection parasite load was measured in spleens and livers from the infected mice by limiting dilution assay.

### Generation of a targeting construct for deletion of the LdUfsp gene

The drug resistance markers nourseothricin was used to obtain LdUfsp^−/−^. To generate the targeting construct, a 793 bp fragment from the 5′ region and an 735 bp fragment from the 3′ region partly overlapping with the LdUfsp open reading frame were amplified by PCR using *L. donovani* genomic DNA. The 5′ flanking fragment included part of the N′ terminal open reading frame (515 bp from the starting methionine) encoding LdUfsp in addition to the UTR. This was done due to the limited amount of 5′ flanking sequence (452 bp) that separates LdUfsp from the upstream ORF. Similarly, the 3′ flanking fragment included part of the LdUfsp open reading frame (385 bp from 1137–1521) encoding LdUfsp due to the limited amount of 3′ flanking sequence (591 bp) that separates LdUfsp from the downstream ORF. The primers used to amplify 5′ flanking fragment included restriction sites HindIII and BamHI. Similarly, the primers added SpeI and XbaI sites to the 3′ flanking fragment. The drug resistance marker nourseothricin was amplified with primers that add BamHI and SpeI to the open reading frame. These DNA fragments were subcloned into the pCR2.1-Topo vector and the nucleotide sequence was determined to ensure fidelity. The plasmid containing the 5′ flanking fragment was digested with HindIII/BamHI, gel purified and ligated into a similarly digested plasmid containing nourseothricin. The resultant plasmid, containing both the 5′flanking region and the drug resistance markers was digested with SpeI/XbaI and the 3′flanking fragment isolated by SpeI/XbaI digestion was ligated into these sites. The authenticity of the final plasmid was confirmed by DNA sequencing. For the purpose of transfection, the targeting construct was prepared by digestion with HindIII/XbaI, which cuts out a linear fragment containing the Ufsp 5′ flanking sequence, the nourseothricin gene and the Ufsp 3′ flanking sequence. The fragment was gel purified and used in transfection.

### Isolation of genomic DNA and Southern blot analysis

Total genomic DNA was isolated from promastigotes with the Wizard genomic DNA purification kit (Promega Biosciences), following the method suggested by the manufacturer. The DNA was digested with restriction endonuclease BglI or XhoI and separated on 1% agarose gels. Southern blot analysis of the resolved DNA was done as described previously using a ^32^P-labelled 620 bp partial LdUfsp coding sequence corresponding to 516–1136 bp of the Ufsp open reading frame as a probe [14].

### Reexpression of LdUfsp in LdUfsp^−/−^ mutant parasites

To restore Ufsp expression in the LdUfsp^−/−^ parasites, the LdUfsp ORF was first PCR amplified using a LdUfsp containing plasmid as template and the following a forward primer: 5′-ACTAGT ATG GAG GAT GTC GTG ACC GGC GTT GC-3′, and a reverse primers: 5′- ACTAGT TCA CTT GTC ATC GTC GTC CTT GTA GTC TCG AGG ATC GAA CAG GTC AAC GCG TGG-3′ that amplify a wild type Ufsp coding sequence or a C327S variant of Ufsp in two independent amplification reactions. These oligos introduced a FLAG epitope tag at the C′ terminus of the recombinant protein. The amplified product was subcloned into the pCR2.1-TOPO cloning vector. The fidelity of the cloned sequence was verified by nucleotide sequencing. The SpeI insert was ligated into the SpeI site of the pKS-Neo vector [20] and the recombinant plasmids, pKSNeo-LdUfsp^WT^ or pKSNeo-LdUfsp^C>S^ was transfected into the LdUfsp^−/−^ promastigotes as described previously [19]. Transfected promastigotes were selected with minimal dose of G418 (20 µg/ml).

### Counting of viable cells

*Leishmania* axenic amastigotes were stained with a solution containing acridine orange and propidium iodide and viable cells were counted using Cellometer instrument.

### Immunofluorescence assay

Immunofluorescence assay was performed essentially as described in [13]. *Leishmania* parasites were fixed in 2% *p*-formaldehyde-PBS for 5 min and allowed to adhere to poly-L-Lysine treated glass slides. After blocking with 5% BSA-PBS, the cells were incubated with either anti-Ufsp antibodies or pre-immune serum. Cellular localization of endogenous Ufsp was detected using anti-rabbit IgG-Alexa488 conjugate antibodies. Nucleic acids were stained with DAPI.

### Cell fractionation

Cell fractionation was performed as described previously [19]. Briefly, *Leishmania* cells were washed three times in 15 ml MES buffer (20 mM MOPS, pH 7.0, 250 mM sucrose, 3 mM EDTA). The cell pellet was resuspended in 0.2 ml MES buffer containing 1 mg/ml digitonin and protease inhibitor cocktail (Roche Applied Science). The suspension was incubated at room temperature for 5 minutes and centrifuged at 10,000 g for 5 minutes. The resulting supernatant was collected as a cytosolic fraction, and the heavy membrane pellet enriched for mitochondria was resuspended in phosphate buffer (20 mM sodium phosphate, pH 7.0, 3 mM EDTA). The polyclonal antibodies against Ufm1, Ufc1, Ufsp, TatD proteins were prepared in our laboratory and were described previously [13], [21].

### Statistical analysis

Student's t-test was used to test the statistical significance of the observed differences.

## Results

### *Leishmania* parasite genomes have a homolog of Ufsp

**Figure 1:**
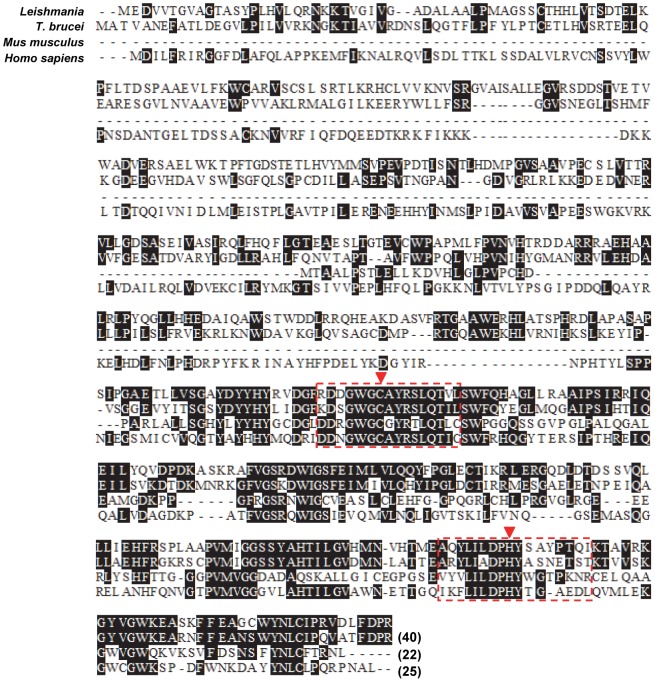
CLUSTAL W alignment of *L. infantum* putative Ufsp with mammalian homologs. The amino acid sequence corresponding to human Ufsp2 was used in this analysis. The asterisks represent identical residues, and dashes represent gaps. The conserved cys and his boxes, containing the conserved blocks of amino acid residues are shown. The amino acid residues important for activity such as cysteine and histidine are indicated with arrows. The percentage identities of human Ufsp2 with those of *L. donovani, T. brucei* and mouse are shown in the parentheses.

Protein modifications mediated by the Ubls in the parasitic organisms such as *Leishmania* are of considerable interest. Our previous studies analyzing the Ufm1 mediated protein conjugation studies in *Leishmania donovani* demonstrated the presence of an Ufm1 conjugation system in this parasite. Further, evidence for an Ufm1 processing reaction to generate a conjugatable Ufm1 by a C- terminal hydrolase activity has been demonstrated in *Leishmania* by the absence of Ufm1 processing when an Ufm1^G>A^ mutant was expressed in *Leishmania* [13]. Therefore, we searched the genome databases of *Leishmania infantum* to find if sequence homologs of human Ufm1 specific proteases such as Ufsp1 (containing 217 amino acid residues) and Ufsp2 (containing 416 amino acid residues) involved in the processing of human Ufm1 [9] are present in *Leishmania*. Studies of human Ufsp have also shown the deconjugation activity of Ufsp i.e., cleaving of Ufm1 from its substrate proteins [9]. Database searches of the *L. infantum* and other trypanasomatid genomes using human Ufm1 processing peptidases as query sequences revealed the existence of a putative Ufm1 specific protease in *L. infantum* (www.tritrypdb.org LinJ.34.3830). Protein sequence alignments using ClustalW (http://www.ebi.ac.uk/Tools/msa/clustalw2/) revealed that the overall amino acid sequence similarity between mouse, human and trypanosomatid Ufsp is relatively low (≤25%) (Fig. 1). However, similar to human deubiquitinating enzymes and Ubl specific proteases, trypanosomatid Ufsp molecules have highly conserved cysteine and histidine residues that form a “Cys box” and a “His box” (Fig. 1 boxed with arrows). Unlike humans that have Ufsp1 and Ufsp2, only a single copy of the Ufsp gene is found in *Leishmania* genome. Structural analysis of the putative *Leishmania* Ufsp protein revealed that it is similar to Ufsp2 of mouse and humans. Together, the sequence homology, conservation of catalytically important amino acid residues and presence of Ufm1 processing activity in *Leishmania* as reported in our previous studies characterizing Ufm1 conjugation associated enzymes [13] indicated that *Leishmania* genome contains a gene encoding Ufsp. Therefore, we explored the biochemical characteristics of Ufm1 processing activity of this putative Ufsp protein encoded in *Leishmania* genome.

### Generation of FRET based activity probes of *Leishmania* Ufm1 and Ufm1^G>A^

**Figure 2:**
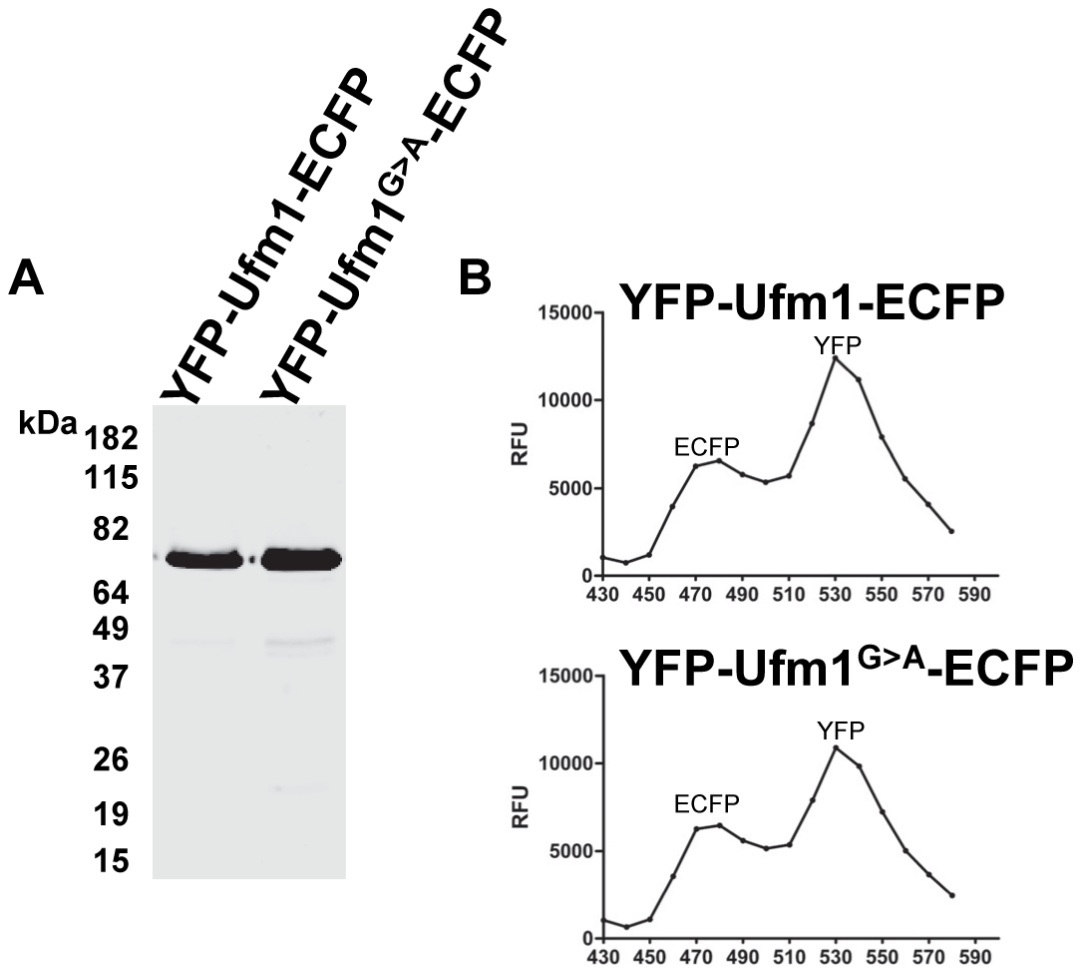
Preparation of FRET activity probes. A) Recombinant proteins encoded by the plasmids YFP-Ufm1-ECFP and YFP-Ufm1^G>A^-ECFP were purified from bacteria. The purified recombinant proteins substrates were run on SDS-PAGE and stained with Gel code blue reagent. B) The fluorescent properties of the recombinant proteins YFP-Ufm1-ECFP and YFP-Ufm1^G>A^-ECFP were verified on a fluorescence plate reader by measuring the emissions from 480 nm to 530 nm with 10 nm intervals upon excitation at 405 nm.

To investigate the protease activity that is responsible for processing the C-terminal extension of Ufm1, we prepared FRET associated activity probes based on the protocol developed by Tatham and Hay [18]. Fluorescence based activity assays offer several advantages over the more commonly used gel based approaches in terms of sensitivity and allow quantitative assessments. Recombinant YFP-Ufm1-ECFP and the mutant version i.e. YFP-Ufm1^G>A^-ECFP were purified from bacteria and SDS-PAGE analysis showed the fusion proteins as a single band on the gel (Fig. 2A). To test the fluorescence properties of these probes and to verify if the recombinant proteins allow the FRET activity to occur, the probes were analyzed on a fluorescent plate reader. Analysis revealed that the fluorescent probes when excited at 405 nm, result in two emission lines; one at 480 nm corresponding to ECFP and another at 530 nm corresponding to YFP (Fig. 2B). Storage of the fluorescent probes at −80°C did not result in measurable deterioration of FRET activity (data not shown).

### Recombinant LdUfsp shows Ufm1 processing activity

**Figure 3:**
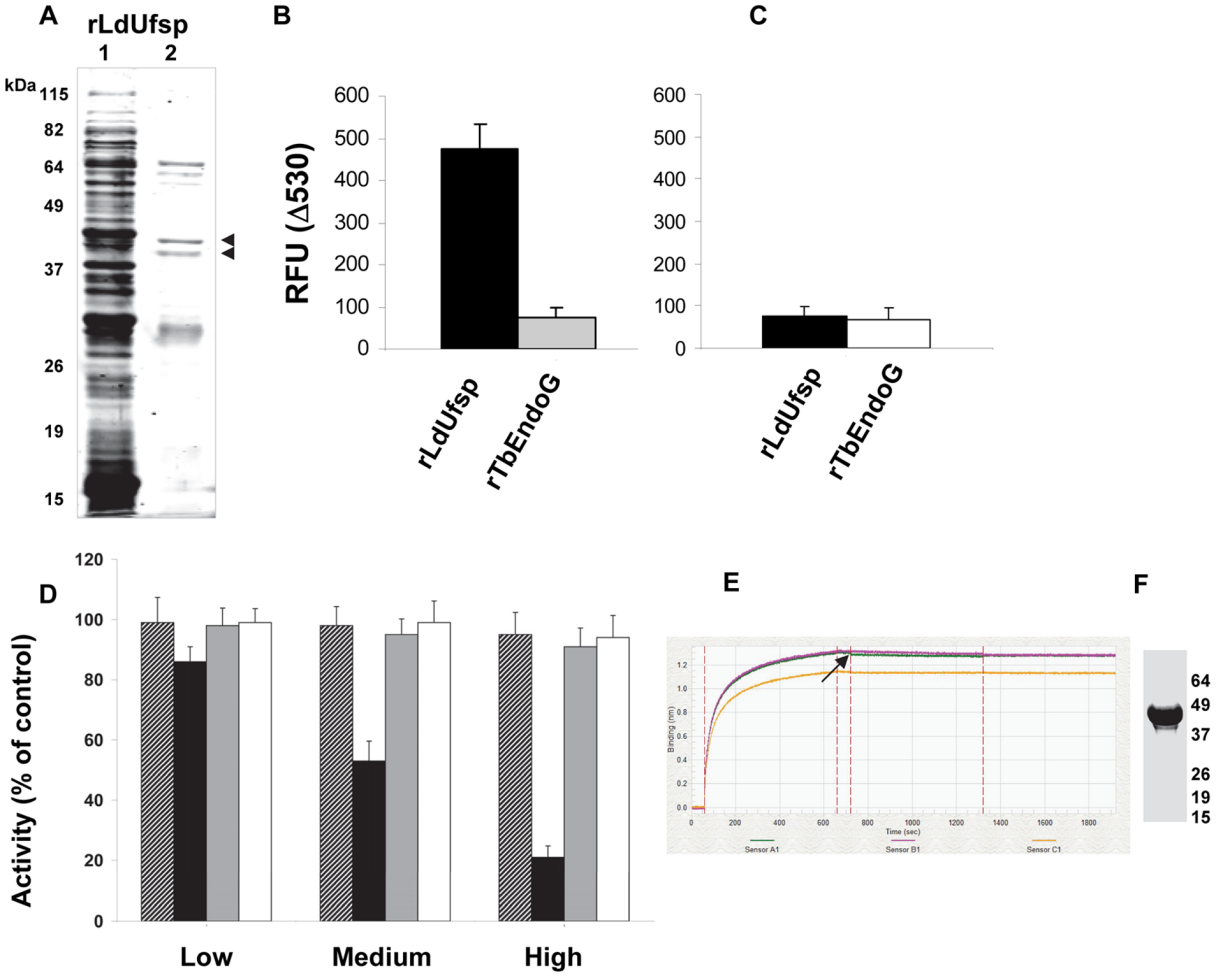
Ufm1 processing activities of the recombinant protein encoded by the putative LdUfsp. A) A recombinant protein encoded by the putative LdUfsp was prepared in bacteria using a partial open reading frame (427–1518 bp) encompassing the regions conserved between the mammalian and trypanosomatid parasites. The recombinant protein is run on the SDS-PAGE and stained with Gel code blue reagent (lane 1 shows the total bacterial lysate and lane 2 the purified recombinant protein; arrow indicates the recombinant protein). B) Ufm1 processing activity of the recombinant protein (rLdUfsp, black bar) was measured by incubation with the fluorescent substrate containing wild type full length Ufm1. In a control reaction, an unrelated protein (rTbEndoG, endonuclease G from *T. brucei*) was tested for the Ufm1 processing activity (grey bar). C) Ufm1 processing activity of the recombinant protein (rLdUfsp, black bar) was measured by incubation with the fluorescent substrate containing Ufm1^G>A^ mutant. As a control, the unrelated protein used above was tested for the Ufm1 processing activity (grey bar). D) Inhibition of Ufm1 processing activity by anti-parasitic drugs. Recombinant Ufsp protein and the fluorescent Ufm1 substrate were incubated in presence of anti-parasitic drugs and the Δ530 values are expressed as % compared to those obtained with 20 mM N-ethyl maleimide. The following doses were used Nifurtimox 1, 10, 100 µg/ml (indicated by the bar with diagonal lines), Amphotericin B 0.1, 1, 10 µg/ml (indicated by the black bar), Melarprosol 0.002, 0.01, 0.2 ng/ml (indicated by the gray bar), and Geramin 0.15, 1.58, 15.8 µg/ml (indicated by the white bar). Low, medium and high represent the doses indicated above. E) Binding of Amphotericin B to biotinylated Ufsp protein measured on a streptavidin sensor on an Octet instrument. Arrow represents shift in the optical interference upon Amphotericin B binding. F) SDS-PAGE gel showing the biotinylated Ufsp protein used in the binding studies.

To test if the recombinant protein encoded by the putative LdUfsp possesses the Ufm1 processing activity, the purified recombinant Ufsp protein was incubated with the fluorescent probes YFP-Ufm1-ECFP and YFP-Ufm1^G>A^-ECFP. Purification of recombinant protein encoded by the partial open reading frame resulted in approximately 43 and 39 kDa bands on a SDS-gel (Fig. 3A, lane 2 indicated by arrow heads). Recombinant human Ufsp2 has been shown to undergo spontaneous cleavage [22] indicating that similar processing might be occurring in LdUfsp (short fragments in Fig. 3A). Ufm1 substrate cleavage results showed that the putative Ufsp protein indeed possesses Ufm1 processing activity (Fig. 3B). This cleavage was not detected in presence of the mutant probe (Ufm1^G>A^) indicating that the cleavage of Ufm1 by the putative Ufsp is specifically occurring at the glycine residue (Fig. 3C). To further verify the specificity of the Ufm1 cleavage activity by the Ufsp, we performed the activity assays using an unrelated recombinant protein (rTbEndoG, a nuclease produced in *E. Coli* [19]) purified in a parallel experiment as a source of the enzyme. Results showed that this unrelated protein did not cleave the fluorescent Ufm1 substrate indicating that any contaminant protein in the bacterial lysate as an unlikely source for the observed Ufm1 cleavage with Ufsp (Fig. 3B, C). To test whether this in vitro Ufm1 processing activity of the recombinant Ufsp can be inhibited by the anti-parasitic drugs that are commonly in use, and thus to verify if Ufsp activity is amenable to inhibition we tested a limited set of the anti-parasitic drugs in our assay. We tested Nifurtimox, Amphotericin B, Melarprosol, and Geramin following the doses most commonly used in in vitro studies [23]–[26]. Results showed that Nifurtimox, Melarprosol, and Geramin did not inhibit Ufm1 processing activity at the concentrations tested (low, medium and high, Fig. 3D). However, Amphotericin B showed inhibitory effects on the Ufm1 processing activity and this inhibition correlated with the dose used (Fig. 3D). To test if the observed inhibition is also accompanied by binding of Amphotericin B to the recombinant Ufsp, we performed binding activity assays using biotinylated Ufsp (Fig. 3 F) on a streptavidin sensor on an Octet instrument. Binding of Amphotericin B to the biotinylated Ufsp produces an optical interference pattern that is recorded by the instrument. Results showed that Amphotericin B can bind to the biotinylated Ufsp protein (Fig. 3E) suggesting a pharmacological basis for the observed inhibitory activity.

### Demonstration of in vivo Ufm1 processing activity by Ufsp

**Figure 4:**
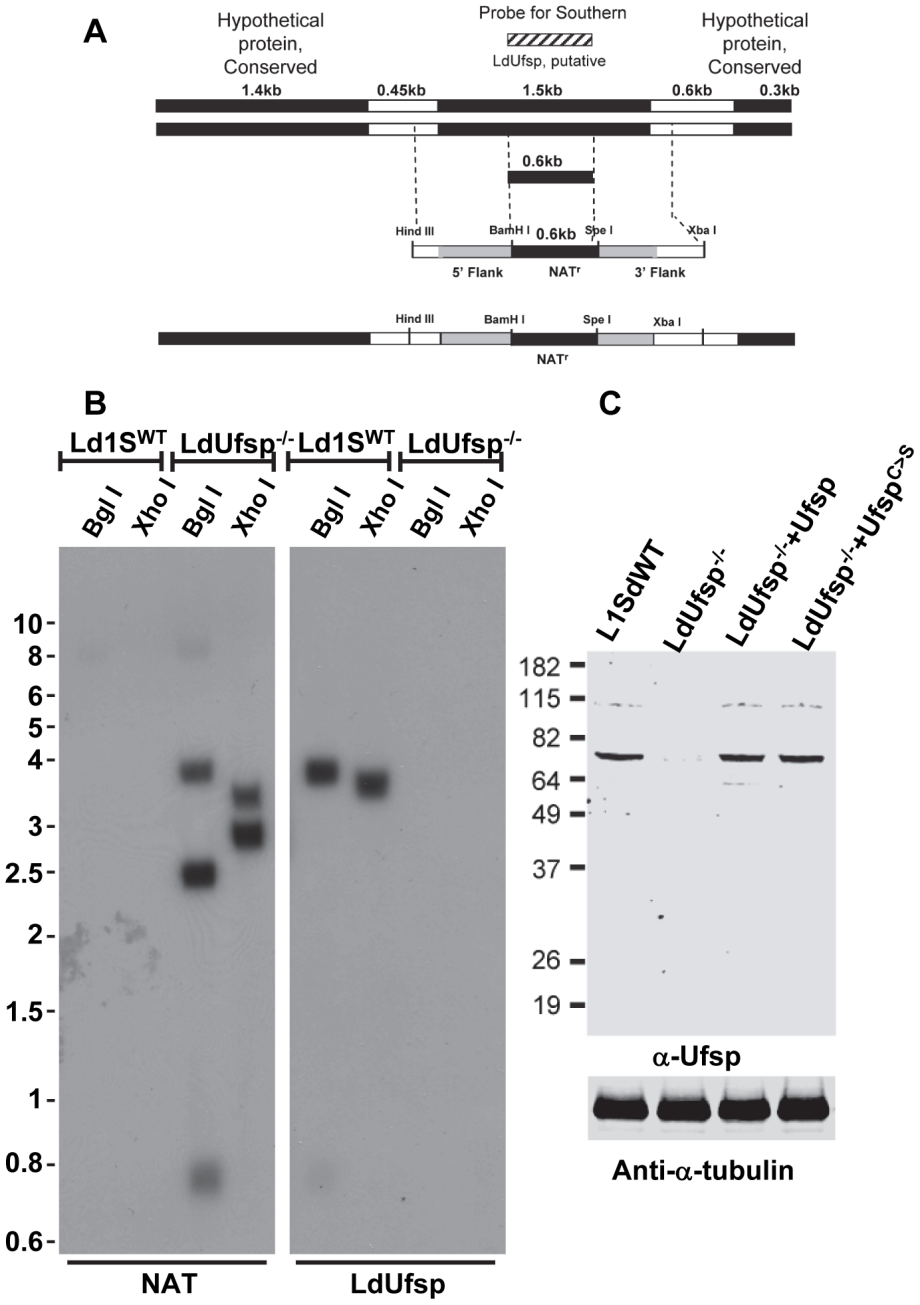
Ufsp gene disruption in the *L. donovani* genome. A. Schematic diagram showing design of construct for LdUfsp gene disruption in the *L. donovani* genome. Nourseothricin construct is flanked on the 5′ and 3′ sides with LdUfsp 5′-UTR (white bar) and 3′-UTR (white bar) and partial open reading frame (grey bar) respectively. The positions of BglI/XhoI restriction sites are indicated. The DNA fragment corresponding to the region targeted for deletion was used as probe on Southern blot (box with diagonal lines). B. Southern blot analysis of the genomic DNA of *Leishmania* wild-type (Ld1SWT) and LdUfsp gene-deleted (LdUfsp^−/−^) parasites with a partial LdUfsp ORF (LdUfsp), and Nourseothricin (NAT) as ^32^P-labelled probes. The genomic DNA from the parasites digested with the restriction enzyme BglI or XhoI was used in the analysis. C. Western blot analysis of the lysates of wild-type (Ld1SWT) and LdUfsp gene-deleted (LdUfsp^−/−^) and parasites reexpressing either wild type (LdUfsp^−/−^+Ufsp^WT^) or a mutant Ufsp (LdUfsp^−/−^+Ufsp^C>S^). The blots were probed with an anti-Ufsp antibody (α-Ufsp) or an anti-α tubulin antibody (anti-α-tubulin) as a loading control.

To investigate if the putative Ufsp is indeed the *in vivo* source for the Ufm1 cleavage activity and to further probe the *in vivo* functions of the Ufsp in *L. donovani*, we generated a *L. donovani* Ufsp null mutant by homologous recombination (Fig. 4A). *L. donovani* promastigotes were transfected by electroporation and the two alleles of LdUfsp were replaced by recombination with a targeting construct containing nourseothricin marker flanked by DNA fragments corresponding to 5′ and 3′ untranslated regions (UTR) of LdUfsp. Southern hybridization of genomic DNA isolated from the LdUfsp null mutants with a ^32^P labeled probe corresponding to the targeted region of Ufsp revealed that both alleles of LdUfsp were lost in the null mutant (Fig. 4B LdUfsp^−/−^lanes probed with LdUfsp) and were replaced by the nourseothricin marker (Fig. 4B LdUfsp^−/−^lanes probed with NAT). Loss of LdUfsp expression in the LdUfsp^−/−^ parasite was confirmed by Western blot analysis using anti-Ufsp antibody (Fig. 4C, LdUfsp^−/−^ lane). This antibody recognizes the full length protein of LdUfsp with a calculated mass of 56 kDa with an apparent mobility of ∼70 kDa (Fig. 4C). LdUfsp expression was restored by transfecting the null mutant cells with the pKSNeo vector containing the coding sequence of either wild type Ufsp or a mutant Ufsp where the catalytic cysteine was altered to a serine. (Fig. 4C, LdUfsp^−/−^+Ufsp^WT^, LdUfsp^−/−^+Ufsp^C>S^). Immunoblotting with an anti-tubulin antibody (anti-chicken tubulin, Sigma) revealed equal loading of protein (Fig. 4C, anti-α-tubulin).

**Figure 5:**
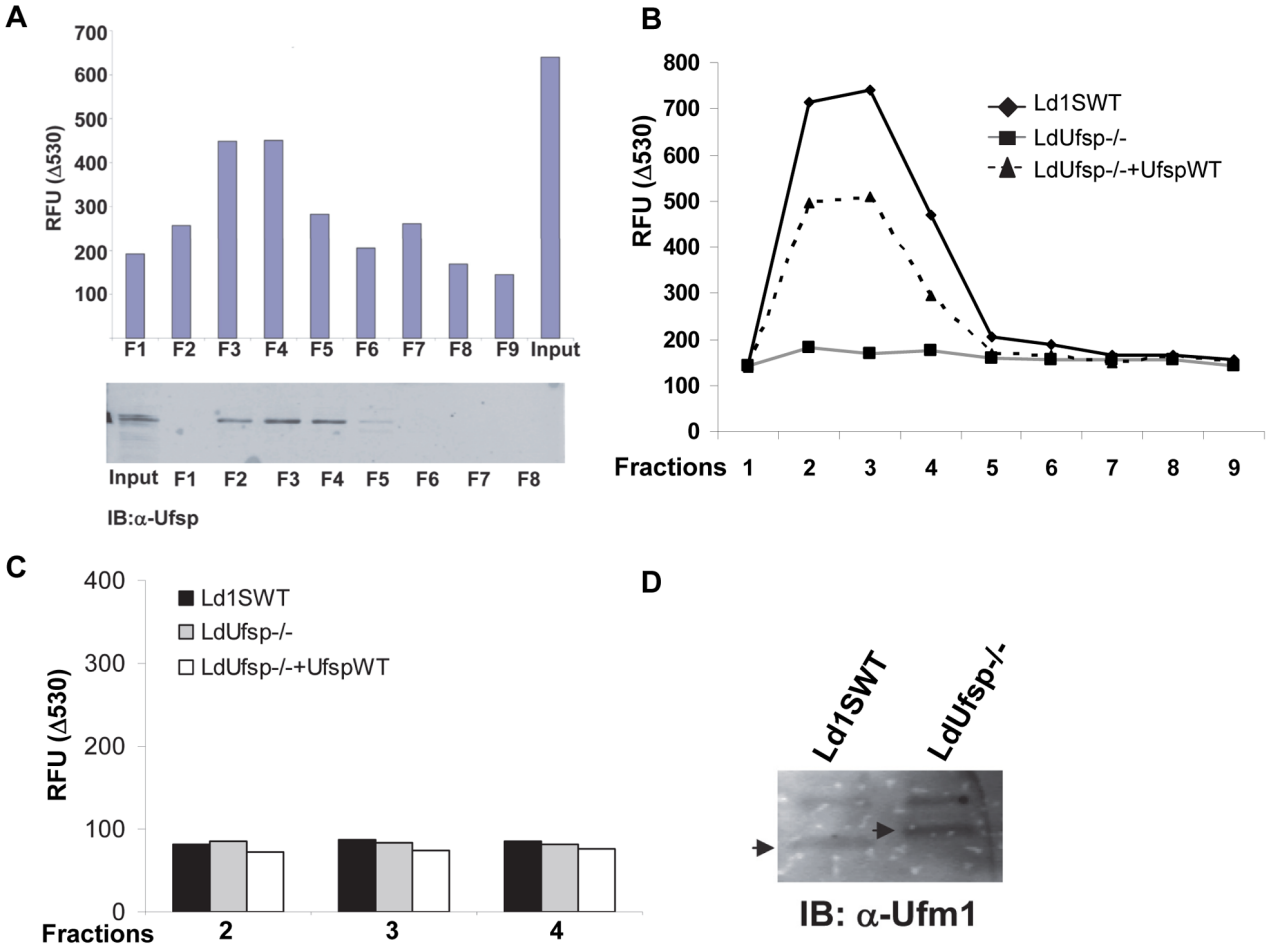
Ufm1 processing activity using fractions derived from Ld1SWT, LdUfsp^−/−^ and add back parasites. A) Ufm1 processing activity of the fractions of the lysates from Ld1SWT cells obtained after fractionation on Q columns were incubated with the fluorescent substrate containing wild type full length Ufm1 substrate (YFP-Ufm1-ECFP). The reduction in the 530 nm fluorescence from the YFP after 60 min incubation is plotted. Immunoblot showing the distribution of Ufsp protein in the fractions is shown. B). Ufm1 processing activity of the fractions of the lysates from Ld1SWT, LdUfsp^−/−^ and LdUfsp^−/−^+Ufsp^WT^ cells obtained after similar fractionation as above were incubated with the fluorescent substrate containing wild type full length Ufm1 substrate (YFP-Ufm1-ECFP). C) Ufm1 processing activity of the fractions of the lysates from Ld1SWT, LdUfsp^−/−^ and LdUfsp^−/−^+Ufsp^WT^ cells obtained after similar fractionation as above were incubated with the mutant Ufm1 substrate (YFP-Ufm1^G>A^-ECFP). Data is representative of three independent experiments. D) Immunoblot of the lysates from Ld1SWT, LdUfsp^−/−^ probed with an anti-Ufm1 antibody.

The *in vitro* biochemical assays using FRET based activity probes indicated that the putative Ufsp has the Ufm1 processing activity. To examine if this Ufsp is indeed the protease responsible for the Ufm1 processing in vivo, we fractionated the cell extracts obtained from the *L. donovani* wild type parasites. For this purpose we utilized fractionation on strong anion columns followed by testing of Ufm1 processing activity. We reasoned that by such fractionation we should be able to concentrate the Ufm1 processing activity into few select fractions reproducibly. Results showed that by using FRET based activity probes we were able to fractionate Ufm1 processing activity from the parasite lysates (Fig. 5A). We resolved the fractions obtained on SDS-PAGE and immunoblot with an anti-Ufsp antibody showed that the protein is enriched in fractions where we observed the peak Ufm1 processing activity (Fig. 5A lower panel). By following an identical fractionation scheme, a comparison of the fractions collected from *L. donovani* wild type, LdUfsp^−/−^ and LdUfsp^−/−^ mutants re-expressing Ufsp should allow us to examine whether *in vivo* Ufm1 processing activity was lost in the LdUfsp^−/−^ mutant. This should also allow us to demonstrate that there are no other cellular sources capable of Ufm1 processing activity. Results showed that fractions from wild type parasites corresponding to 0.3–0.4 M NaCl possess the peak Ufm1 processing activity indicated by the reduction in the 530 nm emission when these fractions were incubated with the YFP-Ufm1-ECFP substrate (Fig. 5B). In contrast, similar fractions from the LdUfsp^−/−^ mutant did not show any reduction in the 530 nm emission indicating the intactness of the YFP-Ufm1-ECFP substrate, thus absence of Ufm1 processing (Fig. 5B). On the other hand, the Ufm1 processing activity was significantly restored in the fractions from the LdUfsp^−/−^ mutants re-expressing Ufsp indicating that indeed Ufsp is the source of Ufm1 processing activity and also that fractionation of exogenously expressed Ufsp follows that obtained from wild type cells.

To further demonstrate that the observed Ufm1 processing activity in the fractions obtained from wild type and Ufsp^−/−^ cells re-expressing Ufsp is specific and dependent on the presence of glycine residue at the C′ terminus of the Ufm1, we performed the Ufm1 processing activity assays with the fractions (Fractions 2, 3 and 4) obtained from the wild type, Ufsp^−/−^ and Ufsp^−/−^ cells re-expressing Ufsp in presence of the mutant probe YFP-Ufm1^G>A^-ECFP (Fig. 5C). Results showed that the cleavage did not occur at a random residue (as there is no glycine at the C′ terminus) in the mutant probe incubated with positive fractions obtained in the previous experiment (Fig. 5B). Further, to test whether the endogenous Ufm1 remains unprocessed in the Ufsp^−/−^ mutant cells and therefore results in a small shift in the molecular weight, we performed an immunoblot from the whole cell lysates prepared from the wild type and Ufsp^−/−^ mutants and probed with an anti-Ufm1 antibody. Results suggested that indeed the Ufm1 remains as unprocessed that is detectable as a slightly higher molecular mass in the Ufsp^−/−^ mutants compared to the wild type cells (Fig. 5D). The slightly higher molecular weights upper band that is reactive to the anti-Ufm1 antibodies is evident in wild type and the Ufsp−/− mutant lanes. This band is identical in both lanes with no apparent shift indicating that this is likely a non-specific reactive band.

### Interaction between Ufsp and Ufm1

**Figure 6:**
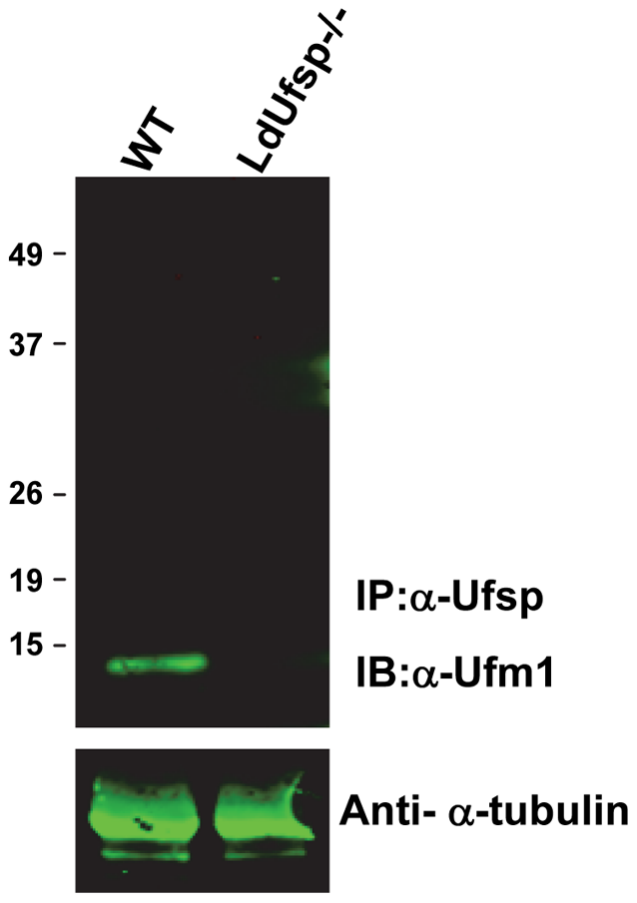
Molecular interaction between Ufsp and Ufm1 in *L. donovani*. Parasite lysates from either wild type (WT) or Ufsp null mutant (LdUfsp^−/−^) were incubated with an anti-Ufsp antibody (IP:α-Ufsp) and the immune complexes were precipitated after over night incubation and resolved on SDS-PAGE. The resulting blots were probed with an anti-Ufm1 antibody (IB:α-Ufm1). The blots were probed with an anti-α-tubulin antibody as control for equal protein load (anti-α-tubulin).

In order to characterize the molecular interaction between Ufm1 and the processing enzyme Ufsp, in *L. donovani*, a co-immunoprecipitation assay was performed with an anti-Ufsp antibody and the blots were probed with an anti-Ufm1 antibody (Fig. 6). Results showed that Ufm1 does interact with Ufsp as revealed by the presence of a ∼12 kDa band corresponding to Ufm1 in wild type *Leishmania* (Fig. 6; WT lane) and not in the Ufsp^−/−^, indicating the specificity of the molecular interaction between Ufm1 and Ufsp proteins (Fig. 6). Together, these results demonstrated that Ufm1 can interact with Ufsp protein. This result is consistent with our previous observation that Ufm1 is processed by the Ufsp in *Leishmania*.

### Immunolocalization of LdUfsp

**Figure 7:**
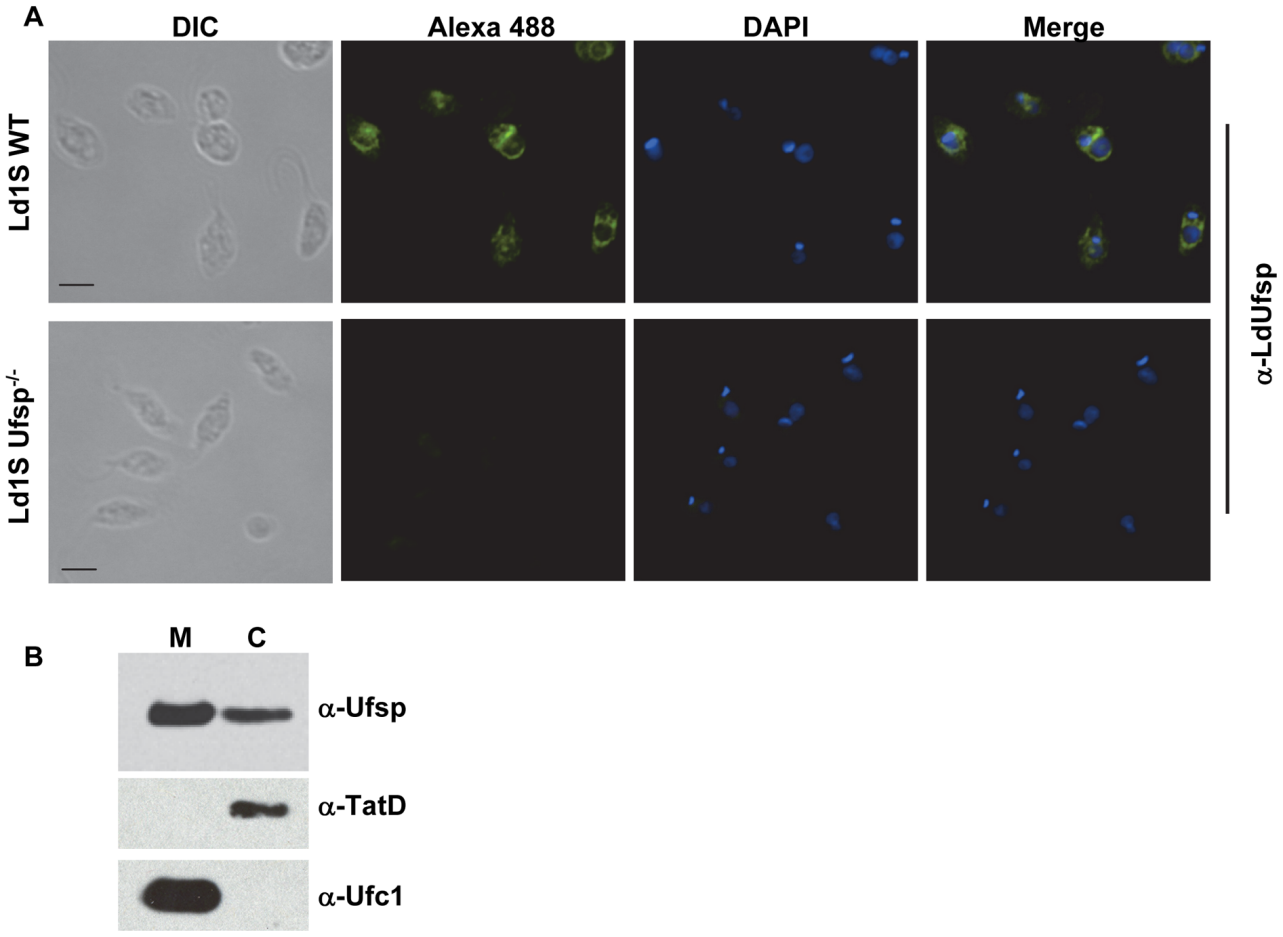
Immunofluorescence analysis of *L. donovani* wild type and LdUfsp^−/−^ parasites. A) Immunofluorescence studies were performed on log-phase amastigote cells (∼1×10^7^/ml) from wild type (Ld1SWT) and Ufsp null mutant cultures (Ld1SUfsp^−/−^). The cells were fixed in 2% paraformaldehyde and incubated with the anti-Ufsp antibody. Anti-rabbit IgG conjugated to Alexa488 was used as a secondary antibody. The nucleic acids were stained with DAPI. The bar represents 5 µm. B) Cell fractionation into mitochondrial (M) and cytosolic (C) fractions followed by immunoblotting with anti-Ufsp, anti-Tat-D (α-Tat-D), a cytosolic marker and anti-Ufc1 (α-Ufc1), a mitochondrial marker.

Previously we have shown that proteins mediating Ufm1 conjugation such as Uba5, Ufc1 as well as a majority of Ufm1 are localized in the mitochondria [13]. Since the Ufm1 processing is the first step in the Ufm1 conjugation cascade, we wanted to investigate the cellular distribution of endogenous LdUfsp protein in *Leishmania*. To this end, antibodies against *L. donovani* Ufsp protein were used in immunofluorescence studies using wild type and Ufsp^−/−^ cells. Immunofluorescence assays revealed that endogenous LdUfsp has a diffuse localization in the cytoplasm of *L. donovani*. (Fig. 7, top panel). The Ufsp−/− mutants did not show any reactivity with the anti-Ufsp antibodies (Fig. 7, bottom panel). No background reactivity was obtained with the pre-immune serum in these experiments (data not shown). To further confirm the localization of the Ufsp in *L. donovani*, we performed cell fractionation studies followed by immunoblotting. Results showed that Ufsp is predominantly localized in the mitochondrial fraction but also present in cytosolic fraction in considerable amount (Fig. 7B lanes M and C). The authenticity of fractionation was confirmed by using antibodies for mitochondrial (Ufc1) and cytosolic (Tat-D) markers. Results showed that Ufc1 and Tat-D markers were only detected in mitochondrial and cytosolic fractions alone respectively (Fig. 7B lanes M and C). These results further suggest that most of the components of *Leishmania* Ufm1 conjugation pathway including Ufsp are localized in mitochondria.

### Loss of Ufsp expression results in reduced parasite growth in vitro, ex vivo and in vivo

**Figure 8:**
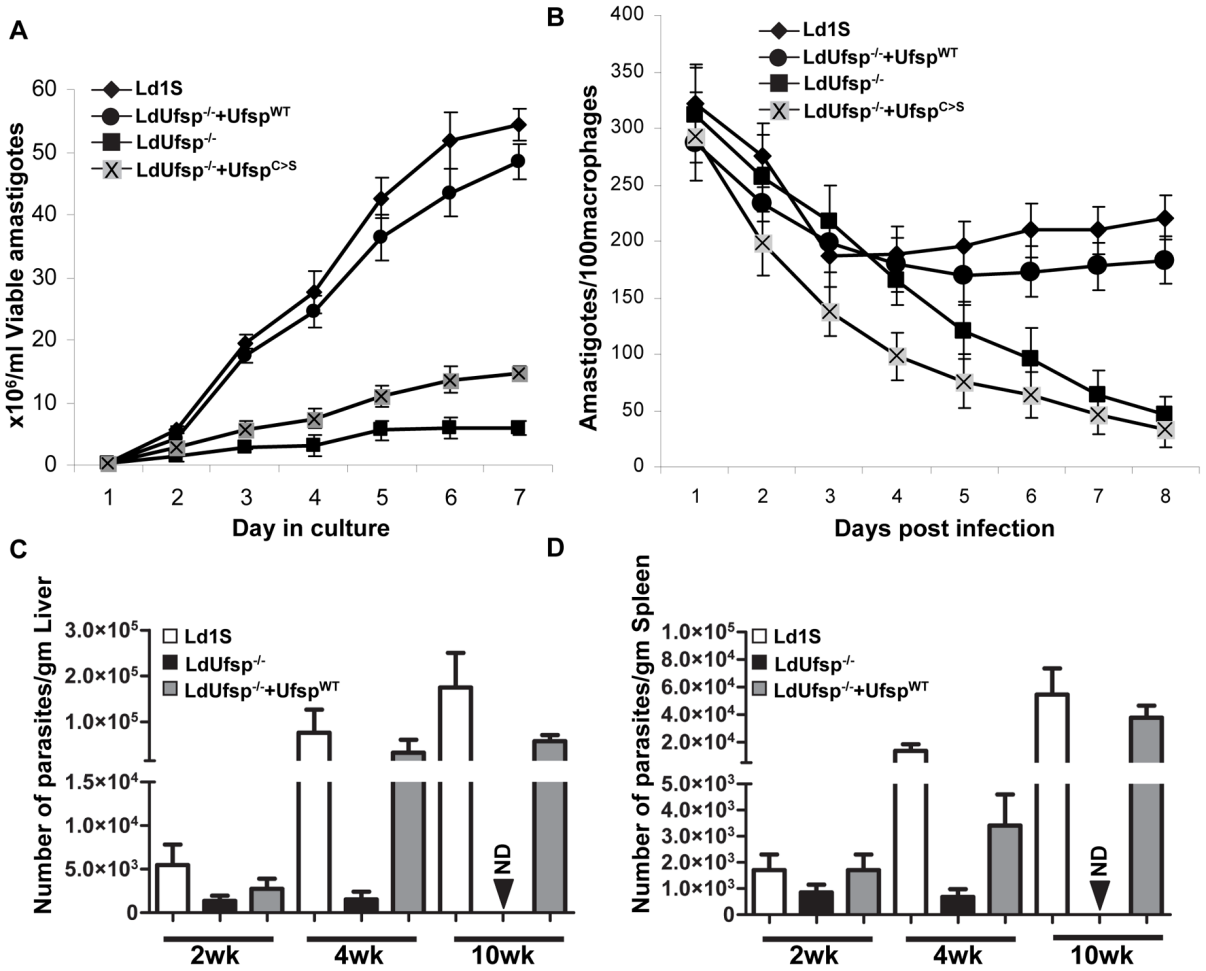
Effects of Ufsp deletion on the survival of *L. donovani* amastigotes. (A) The growth of *L. donovani* wild-type (Ld1S), or Ufsp null mutant (LdUfsp^−/−^) or mutants reexpressing either wild type (LdUfsp^−/−^+Ufsp^WT^) or mutant Ufsp (LdUfsp^−/−^+Ufsp^C>S^) proteins was monitored in axenic amastigote culture. (B) Human macrophages differentiated from monocytes were infected with stationary phase promastigote parasites from *L. donovani* wild type (Ld1S), Ufsp null mutant (LdUfsp^−/−^) or mutants re-expressing either wild type (LdUfsp^−/−^+Ufsp^WT^) or mutant Ufsp (LdUfsp^−/−^+Ufsp^C>S^) for six hours (10∶1 parasite-to-macrophage ratio) and the numbers of amastigotes in these cultures were determined over a period of 6 days by microscopic observation of Diff-quik reagent stained slides. The data are expressed as the number of amastigotes per 100 macrophages. Error bars indicate the standard deviation. The results are the mean of three independent experiments. Survival of *L. donovani* wild-type (Ld1S), or Ufsp null mutant (LdUfsp^−/−^) or mutants reexpressing wild type (LdUfsp^−/−^+Ufsp^WT^) in liver (C) and spleen (D) of BALB/c mice. Mice were infected with *L. donovani* parasites and at 2, 4, and 10 wk post infection parasite load in infected mice was determined. The numbers represent number of parasites/gram tissue of liver and spleen.

We have previously shown that Ufm1-deficient *L. donovani* parasites show defects in fatty acid metabolism in the amastigote stage of the parasite and since the fatty acid metabolism is necessary for energy generation, we wanted to analyze what effect loss of Ufm1 processing in the Ufsp^−/−^ mutants have on growth *in vitro* as well as ex vivo in human macrophages. Survival of the axenic amastigotes was analyzed by counting viable cells over a period of 7 days (Fig. 8A). The data showed that the Ufsp^−/−^ parasites failed to grow as amastigotes after 3 days in culture (Fig. 8A). Re-expression of wild type Ufsp restored the growth (Fig. 8A). The growth was not restored when a mutant Ufsp (Ufsp^C>S^) was expressed in the Ufsp^−/−^ background (Fig. 8A). The growth of promastigotes was not affected in Ufsp−/− mutants (data not shown). Next, we examined their growth in macrophages ex vivo. To this end, in vitro differentiated human macrophages were infected with stationary phase cultures of wild type, LdUfsp^−/−^ and Ufsp re-expressing either wild type (Ufsp^WT^) or mutant (Ufsp^C>S^) promastigotes (Fig. 8B). The results at 6 h post-infection showed that the percentage of macrophages that are infected with the parasites was similar with all four cell types. These macrophage cultures were subsequently examined at 1, 2, 3, 4, 5, 6 and 7 days post-infection, and the percentage of infected macrophages was calculated. After 4 days the Ld Ufsp^−/−^ and Ld Ufsp^−/−^+Ufsp^C>S^ expressing cells start to show significant decline in growth (Fig. 8B), whereas wild type control cells and add-back cells with wild type LdUfsp continue to grow inside macrophages (Fig. 8B). By day 6, LdUfsp^−/−^ and Ld Ufsp^−/−^ with Ufsp^C>S^ expressing cells dropped to <1 parasite/macrophage. To further test the virulence of LdUfsp^−/−^ parasites in vivo, we conducted mouse infection experiments and measured the parasite burdens at defined time intervals. Results showed that deletion of Ufsp leads to reduced parasite growth and by 10 weeks post infection, parasite burdens reached to non-detectable levels in LdUfsp^−/−^ infected liver and spleens (Fig. 8C and D) whereas in the mutants parasites in which Ufsp is added back (Ufsp^−/−^+Ufsp^WT^) the parasite growth reached comparable levels to that of wild type parasites. These results indicate that lack of Ufm1 processing in LdUfsp^−/−^ could result in growth reduction in amastigotes as was observed by us in the Ufm1^−/−^ parasites [14].

## Discussion

Ubiquitin-like protein modifiers (Ubls) although share the β-grasp fold structure among the various members, regulate a strikingly broad set of cellular processes including proteolysis, endocytosis, membrane trafficking, protein kinase activation, DNA repair, autophagy and chromatin dynamics in eukaryotic cells [4], [27]. The dynamic process of addition and removal of ubl molecule to its substrates is widely conserved throughout in evolution in organisms from yeast to humans and more recently demonstrated even in prokaryotes [28]. Therefore investigation of the various Ubls and their functions in parasitic protozoa assumes importance as identification of ubl conjugation and deconjugation reactions uniquely found in the parasites might provide novel targets for developing inhibitors that block these activities. Inhibition of LdUfsp by Amphotericin B in our studies is of particular interest since this demonstrates the feasibility of developing novel inhibitors against Ufsp. However, there are serious side effects of this drug treatment in patients. Since our results showed the binding of the drug to Ufsp, inhibition of Ufsp activity and essential nature of this activity for *Leishmania* pathogenesis it will be important to explore whether non-toxic regimens of Amphotericin B such as liposomal form of Amphotericin B [29] can also inhibit this activity and molecules analogous to Amphotericin B could be tested as inhibitors against Ufsp to support their use in treatment. Ufm1 in *Leishmania* is unusual in its structure compared to the mammalian Ufm1. *Leishmania* Ufm1 has a 17 amino acid long C′ terminal extension where as the human homolog has only 2 residues beyond the C′ terminal glycine implies that the active sites of the Ufm1 processing enzyme from mammalian and *Leishmania* are likely to be different in terms of their three dimensional conformation. Such possibility makes further studies on *Leishmania* Ufsp structure function highly attractive. Low overall homology between human and *Leishmania* Ufsp proteins (∼25% Fig. 1) makes it feasible to identify small molecular compounds that selectively inhibit parasitic Ufsp activity and could help in development of novel drugs. Previous studies in *Plasmodium* have demonstrated the utility of such approaches in identifying subtle differences in the plasmodium specific ubiquitin and Nedd8 hydrolase UCHL3 enzymatic activities [30]–[32] that may be amenable to inhibition by novel inhibitors. More recently, *Plasmodium* SUMO-specific protease (PfSNEP1) has been shown to possess a unique cleavage sequence preference compared to human SUMO-specific protease and this difference was exploited in identifying inhibitors specific to *Plasmodium* [33]. Such studies in *Leishmania* parasites are warranted since the parasites belonging to *Leishmania* species cause considerable mortality and resistance against the available drugs renders the therapies ineffective.

Previous studies to identify the Ubl processing protease activities relied on chemically modifying the Ubl into an electrophile in an active-site directed reaction and co-precipitating the processing activity using this probe followed by identification by proteomic methods [9], [34]. In the present study we utilized genetic methods to generate an Ufsp null mutant that not only allowed us to demonstrate the Ufm1 processing activity biochemically but also allowed us to investigate the consequence of this deletion to the parasite survival. Deletion of Ufsp resulted in abolition of Ufm1 processing demonstrating that Ufsp is specific to Ufm1 and other protease activities can not compensate for the loss of Ufsp. This is also demonstrated by the re-expression experiments in which the growth of the *Leishmania* amastigotes is restored only when a wild type Ufsp is expressed but not in case of the Ufsp^C>S^ mutant. This is consistent with the absence of Ufm1 processing activity in the Ufsp^C>S^ mutant suggesting that a functional Ufm1 processing activity is necessary for the survival of the parasite.

Ubls such as Ubiquitin, SUMO and NEDD have been identified in medically important parasitic protozoa and are being investigated in these organisms [30], [31]. Recently, Ubl targets that are unique to parasitic protozoa have been described. For instance, several proteins including metacaspase-3, thymidine hydroxylase and histone acetyl transferase were found to be potential sumoylation targets in *Trypanosoma cruzi* [35], [36]. Arguably, sumoylation of thymidine hydroxylase and histone acetyl transferase, DNA and chromatin modification proteins might represent regulation of transcriptional activity in these parasites. Similarly, SUMOylation of paraflagellar rod protein has been shown to be essential for flagellar homeostasis in *T. cruzi* [37]. These unique modifications by SUMO might represent novel functions in the protozoan parasites such as *T. cruzi*.

Previously we demonstrated the existence of Ufm1 conjugation pathway in *L. donovani* [13]. To understand the significance of post-translational modifications in regulating *Leishmania* differentiation mediated by the Ufm1 conjugation, we generated Ufm1 knockout mutant parasites, LdUfm1^−/−^ and characterized the function of the Ufm1 pathway and its role in *Leishmania* pathogenesis [14]. Deletion of Ufm1 from *L. donovani* resulted in loss of β-oxidation of fatty acids due to the absence of conjugation of MTP with Ufm1 [14]. Amastigote stage specific growth defects in the Ufm1^−/−^ studies suggested the importance of conjugation in amastigote stage thus Ufm1 C-terminal processing activity of the enzyme Ufsp might be an important target for inhibition. In this report, we demonstrated the Ufm1 processing activity of LdUfsp both biochemically and by genetic analysis and that LdUfsp is the cellular source of Ufm1 processing activity. LdUfsp^−/−^ parasites grow normally as promastigotes but are growth defective in the amastigote stage, suggesting the importance of LdUfm1 mediated conjugation in the virulent form of the parasite. LdUfsp−/− parasites showed reduced survival in macrophages and also in Balb/C mice that are highly susceptible to *Leishmania* infection. Metabolic deficiency in the β-oxidation of fatty acids previously observed in LdUfm1−/− parasites coupled with the observation that no compensatory activity for the absence of Ufsp is apparent in these mutants suggests that reduced survival of Ufsp−/− parasites could be primarily due to intrinsic defects in the mutant parasites likely in the cell division by altering the Ufm1 function which has an indirect effect on the function of Ufsp protein [14]. However, enhanced parasite killing due to anti-microbial activities of macrophages such as increased NO production could also be contributing to the elimination of the Ufsp−/− mutants from the infected mice.

This is consistent with our earlier observations where we showed that Ufm1 null mutant had amastigote stage specific growth defects [14]. In our previous studies, we have shown that Ufm1, Uba5 and Ufc1 are localized to the mitochondria [13]. The presence of Ufsp in mitochondria further supports the idea that Ufm1 pathway is of mitochondrial origin in *Leishmania*.

In conclusion, our study demonstrates for the first time role of the Ufm1 processing enzyme Ufsp in *Leishmania* pathogenesis that has not been described in other organisms. Studies of Ubl mediated modifications in *T. cruzi*, *T. brucei* and *L. donovani* show evidence that ubl mediated modifications can be parasite specific and distinct from those observed in human Ubls. Studies of Ufsp mediated modifications in parasitic protozoa are likely to lead to a deeper understanding of their contribution to parasite pathogenesis. Further, since Ufsp deficiency does not affect promastigote stages, other components of the Ufm1 conjugation system such as Uba5 and Ufc1 may also be targets for developing novel inhibitors provided their unique functions in Ufm1 conjugation are demonstrated conclusively. It is of particular importance that growth of an Ufsp knock out mutant is attenuated in the amastigote stages; as such stage specific attenuation makes it a promising candidate to be tested as a live attenuated vaccine. Moreover, specific inhibition of Ufsp activity by antileishmanial compound raises the possibility that this enzyme can be exploited as a drug target. In addition, the studies described here with Ufsp in *Leishmania* could help in the understanding of its function in other organisms.
